# IL36G is associated with cutaneous antiviral competence in psoriasis

**DOI:** 10.3389/fimmu.2022.971071

**Published:** 2022-09-12

**Authors:** You-Wang Lu, Yong-Jun Chen, Nian Shi, Lu-Hui Yang, Hong-Mei Wang, Rong-Jing Dong, Yi-Qun Kuang, Yu-Ye Li

**Affiliations:** ^1^ Department of Dermatology and Venereology, First Affiliated Hospital of Kunming Medical University, Kunming, China; ^2^ Hubei Provincial Key Laboratory of Occurrence and Intervention of Kidney Diseases, Medical College, Hubei Polytechnic University, Huangshi, China; ^3^ Department of Dermatology, Huangshi Central Hospital, Affiliated Hospital of Hubei Polytechnic University, Edong Healthcare Group, Huangshi, China; ^4^ NHC Key Laboratory of Drug Addiction Medicine, First Affiliated Hospital of Kunming Medical University, Kunming Medical University, Kunming, China; ^5^ Scientific Research Laboratory Center, First Affiliated Hospital of Kunming Medical University, Kunming, China

**Keywords:** psoriasis, antiviral protein (AVP), IL36G, keratinocyte, treatment

## Abstract

**Background:**

Psoriasis is a common inflammatory skin disease that has a great impact on patients’ physical and mental health. However, the causes and underlying molecular mechanisms of psoriasis are still largely unknown.

**Methods:**

The expression profiles of genes from psoriatic lesion samples and skin samples from healthy controls were integrated *via* the sva software package, and differentially expressed genes (DEGs) between psoriasis and healthy skin were screened by the limma package. Furthermore, GO and KEGG pathway enrichments for the DEGs were performed using the Clusterprofiler package. Protein–protein interaction (PPI) networks for the DEGs were then constructed to identify hub genes. scGESA analysis was performed on a single-cell RNA sequencing dataset *via* irGSEA. In order to find the cytokines correlated with the hub genes expression, single cell weighted gene co-expression network analyses (scWGCNA) were utilized to build a gene co-expression network. Furthermore, the featured genes of psoriasis found in suprabasal keratinocytes were intersected with hub genes. We then analyzed the expression of the intersection genes and cytokines in the integrated dataset. After that, we used other datasets to reveal the changes in the intersection genes’ expression levels during biological therapy. The relationship between intersection genes and PASI scores was also explored.

**Results:**

We identified 148 DEGs between psoriatic and healthy samples. GO and KEGG pathway enrichment analysis suggested that DEGs are mainly involved in the defense response to other organisms. The PPI network showed that 11 antiviral proteins (AVPs) were hub genes. scGSEA analysis in the single-cell transcriptome dataset showed that those hub genes are highly expressed in keratinocytes, especially in suprabasal keratinocytes. ISG15, MX1, IFI44L, and IFI27 were the characteristic genes of psoriasis in suprabasal keratinocytes. scWGCNA showed that three cytokines—IL36G, MIF, and IL17RA—were co-expressed in the turquoise module. Only interleukin-36 gamma (IL36G) was positively correlated with AVPs in the integrated dataset. IL36G and AVPs were found co-expressed in a substantial number of suprabasal keratinocytes. Furthermore, we found that the expression levels of IL36G and the 4 AVPs showed positive correlation with PASI score in patients with psoriasis, and that these levels decreased significantly during treatment with biological therapies, but not with methotrexate.

**Conclusion:**

IL36G and antiviral proteins may be closely related with the pathogenesis of psoriasis, and they may represent new candidate molecular markers for the occurrence and severity of psoriasis.

## Introduction

Psoriasis is a common immune-mediated inflammatory dermatosis, prevalent worldwide, that can have serious effects on patients’ physical and mental health (1). The pathogenesis of psoriasis depends on genetic susceptibility, infection, immune abnormalities, and psychoneurotic factors, resulting in chronicity and leaving patients prone to the recurrence of erythematous plaques with adherent silvery scales (2). The main pathological characteristics of psoriatic lesions are hyperproliferation and aberrant differentiation of epidermal keratinocytes. Even though much is known about this condition, the multifactorial nature of its pathogenesis has not been studied thoroughly. In particular, the extent of the role infection plays in the pathogenesis of psoriasis remains controversial.

A previous study found that persistent subclinical streptococcal and staphylococcal infections may be responsible for not only recurrent acute guttate psoriasis, but also new episodes of chronic plaque psoriasis (3). Recent studies have demonstrated that the pathophysiology of psoriasis is closely associated with antimicrobial peptides (AMPs), including cathelicidin (LL-37), human β-defensins, S100 proteins, and lipocalin 2 (4–6). AMPs are an essential part of the innate immune system’s defense against pathogen-based infections (7). Furthermore, it has been confirmed that infection is involved in the pathological mechanism of psoriasis. Levels of keratinocyte-derived antiviral proteins (AVPs) of 2–5-oligoadenylate synthase 2 (OAS2) were found to be significantly elevated in both the epidermis and serum of psoriasis patients (8). Gao et al. found that AVPs, such as ISG15, MX1, OAS2, OASL, and OAS3, were overexpressed in cultured human keratinocyte cells (HaCaT) stimulated by tumor necrosis factor-alpha (TNF-α) in a psoriasis cell model (9). Moreover, ISG15, RSAD2, IRF7, MX2, and TRIM22 have been found to be overexpressed in psoriatic skin. However, another study found no increased expression of AVPs in skin affected by atopic dermatitis, a chronic inflammatory disease similar to psoriasis (10). It is therefore of interest to determine the link between skin antiviral phenotype and the pathogenesis of psoriasis. Therefore, it is imperative to explore the roles of AVPs in the occurrence of psoriasis.

Herein, we merged five mRNA microarray datasets from the gene expression omnibus (GEO) database to screen for differentially expressed genes (DEGs) between psoriasis and healthy samples. Subsequently, enrichment analysis and protein–protein interaction (PPI) network analysis were performed to screen for the hub gene. We then explored the underlying mechanism of hub genes in a single-cell transcriptome. The workflow for this study is shown in **Figure S1**.

## Material and methods

### Data sources and preprocessing

We obtained the psoriasis transcriptome dataset from GEO, which included the GSE13355 dataset (58 psoriatic lesion samples and 58 non-lesion samples), the GSE30999 dataset (85 psoriatic lesion samples and 85 non-lesion samples), the GSE34248 dataset (14 psoriatic lesion samples and 14 non-lesion samples), the GSE41662 dataset (24 psoriatic lesion samples and 24 non-lesion samples), the GSE14905 dataset (33 psoriatic lesion samples and 28 non-lesion samples), and the GSE162183 dataset (including the lesions of 3 patients and similar regions from 3 healthy donors). Besides these datasets, the GSE85034 (30 psoriatic lesion tissues before and after adalimumab or methotrexate (MTX) treatment), GSE51440 (39 psoriatic lesion tissues before and after guselkumab treatment), GSE117468 (41 psoriatic lesion tissues before and after brodalumab treatment and 44 psoriatic lesion tissues before and after ustekinumab treatment), GSE41664 (36 psoriatic lesion tissues before and after etanercept treatment), and GSE69967 (26 psoriatic lesion tissues before and after tofacitinib treatment) datasets are also included in our study (**Table 1**).

**Table 1:** The GEO datasets included in the present study.

GEO accession	Diagnosis	Treatment	Lesional skin	Non-lesional	Platforms	Severity	PubMed ID
GSE13355	psoriasis vulgaris	None	58	58	GPL570	NA^*^	19169254
GSE30999	psoriasis vulgaris	None	85	85	GPL570	moderate to severe	22763790
GSE34248	psoriasis vulgaris	None	14	14	GPL570	NA	23308107
GSE41662	psoriasis vulgaris	None	24	24	GPL570	moderate to severe	23308107
GSE14905	psoriasis vulgaris	None	33	28	GPL570	NA	18648529
GSE162183	psoriasis vulgaris	None	3	3	GPL24676	NA	33958582
GSE85034	psoriasis vulgaris	Adalimumab 16 weeks	17	17	GPL10558	moderate to severe	27667537
GSE85034	psoriasis vulgaris	MTX 16 weeks	13	13	GPL10558	moderate to severe	27667537
GSE117468	psoriasis vulgaris	Brodalumab 12 weeks	41	41	GPL570	moderate to severe	31883845
GSE117468	psoriasis vulgaris	Ustekinumab 12 weeks	44	44	GPL570	moderate to severe	31883845
GSE41664	psoriasis vulgaris	Etanercept 12 weeks	36	36	GPL570	moderate to severe	23308107
GSE69967	psoriasis vulgaris	Tofacitinib 12 weeks	26	26	GPL570	moderate to severe	27059729

*NA, not available.

All patients whose samples were included in this study were diagnosed with psoriasis vulgaris. The list of cytokines was obtained from https://www.immport.org/resources/cytokineRegistry. During data integration, when a given gene expression was detected across all five datasets, the genes were preserved for further analysis. The expression profiles were integrated *via* the Combat function of the sva package (11) to remove the batch effects.

### Analysis of differentially expressed genes

The DEGs between psoriasis and normal tissues were identified by the limma R software package. Moreover, |log2 fold change (FC) |≥ 2, with an adjusted *p*-value of < 0.05, was set as the threshold for differentially expressed genes selection.

### GO and KEGG pathway enrichment analyses

The Gene Ontology (GO) and Kyoto Encyclopedia of Genes and Genomes (KEGG) enrichment analyses were performed using the clusterProfiler software package (v 4.4.1) to explore the biological function of the DEGs. GO terms of the KEGG pathway with a false discovery rate (FDR) < 0.05 were considered to be significantly enriched.

### Construction of the protein–protein interaction network and hub gene screening

The DEGs were imported into the STRING protein database (STRING11.5; https://string-db.org/) with a combined score >0.9 was adjusted to obtain the protein–protein interaction network. The plug-in CytoHubba (http://apps.cytoscape.org/cytohubba) was used to calculate the topological characteristic parameters of the nodes in the PPI network to screen out the hub genes. The degree method was used to explore the hub genes in the PPI network, and the screening condition was degrees ≥ 10.

### Data processing for single-cell RNA sequencing

The GSE162183 expression matrices were obtained from the GEO database. Quality control and dimensionality reduction were performed by the Seurat software package (v4.1.1). For the initial QC step, we created Seurat objects for the normal and psoriasis groups and filtered out the cells that expressed < 200 genes. Genes expressed in fewer than 3 cells were also excluded. I gene expression profiles of the remaining cells were then normalized, and 2,000 highly variable genes from each sample were identified by the vst method. All genes were scaled, and the principal component analysis was conducted. The cells were clustered by unsupervised clustering (resolution = 0.5) and visualized by umap using the top 20 principal components. Cell-type annotation was performed using the singleR software package (v 1.8.1) and refined with manual annotation. Integration of all single-cell rank-based gene set enrichment analyses with the AVPs was performed by irGSEA (v 1.1.2), and the calculation of the enrichment scoring method was set as UCell.

### Single-nucleus consensus weighted gene coexpression network analysis

Single-nucleus consensus weighted gene coexpression network analysis (scWGCNA) is a systematic biological method used to construct a scale-free network based on single-cell gene expression profiles. In the present study, scWGCNA was performed on the expressions and clinical prototypes found in the GSE162183 dataset. First, we constructed metacells with *k* = 200, then a signed similarity matrix was created. The signed similarity matrix was then raised to power β = 16, varying between cell types, to emphasize strong correlations and reduce the emphasis of weak correlations on an exponential scale. The resulting adjacency matrix was then transformed into a topological overlap matrix. Modules were defined using specific module-cutting parameters, including a minimum module size of 25 genes, a deepSplit score of 2, and a merge Cut Height of 0.6. Module eigengenes (MEs) were identified using the package’s ME function, and signed module membership (MM) was utilized to determine the correlation of MEs with individual genes. Gene significance (GS) was defined as the correlation between the gene expression level and the psoriasis. Modules with the strongest positive correlations with psoriasis were selected.

### Statistical analysis

All statistical analyses were performed on R software (v4.1.2). Differences between groups were compared by the Mann-Whitney-Wilcoxon test. The correlation between immune infiltrating cells and core genes was measured by Pear’on’s chi-square test. An adjusted *p*-value of < 0.05 was considered statistically significant.

## Results

### Differential gene expression analysis

**Figure 1:**
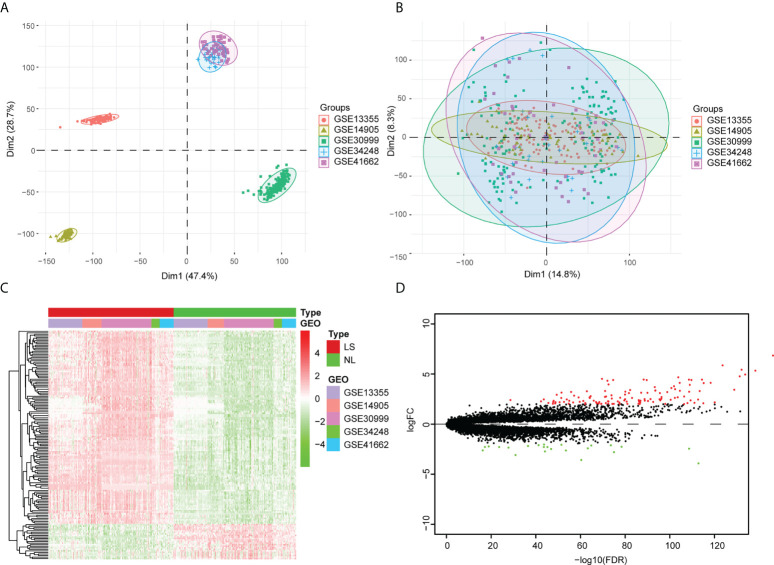
Analysis of the differentially expressed genes within the integrated dataset. **(A)** Principal component analysis (PCA) before batch effect adjustment. Samples from the different datasets cluster together. **(B)** PCA after batch effect adjustment. Samples from different datasets overlap. Red points: samples from GSE1335. Olive green points: samples from GSE14905. Spring green points: samples from GSE30999. Blue points: samples from GSE34248. Pink points: samples from GSE41662. **(C)** The DEG Volcano map shows genes in psoriatic lesion samples (LS) and non-lesion samples (NL). Up-regulated genes are in red; down-regulated genes are in green. **(D)** DEG expression heat map of skin tissue, with high expression in red and low expression in green.

The GSE13355, GSE30999, GSE34248, GSE41662, and GSE14905 datasets were merged into an integrated dataset, which contained 214 psoriatic lesion samples (LS) and 209 non-lesion samples (NL). Before the adjustment, the clustering of samples was largely driven by batch effect, which is due to merging the data from different datasets (**Figure 1A**). After applying ComBat, the batch effect was mitigated (i.e., samples from different datasets were mixed together) (**Figure 1B**). The gene differential expression in the integrated dataset was analyzed *via* the limma package. Based on standard threshold values for |log2 fold change (FC) |≥ 2, with an adjusted *p*-value of < 0.05, 148 DEGs were identified, 125 of which were up-regulated and 23 of which were down-regulated (**Figures 1C, D**) (**Table S1**).

### Pathway enrichment analysis and protein–protein interaction analysis

**Figure 2:**
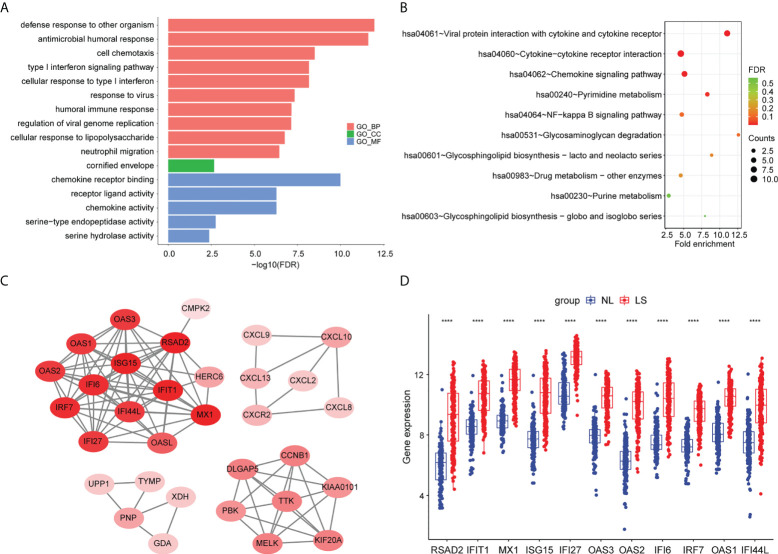
Pathway enrichment analyses and PPI network of DEGs. **(A)** Bar chart of differentially expressed genes’ GO enrichment analysis. **(B)** Bubble chart of differentially expressed genes’ KEGG enrichment analysis. The top 10 pathway enrichments are shown. **(C)** The PPI network of the DEGs. **(D)** Hub genes’ expression between psoriatic lesion samples (LS) and non-lesion samples (NL). p values were calculated using a Wilcoxon signed-rank test. Asterisks corresponding to p values<0.05 (****< 0.0001).

To further investigate the biological functions of the DEGs, we performed a pathway enrichment analysis. The result of the GO enrichment analysis showed that DEGs were mainly related to defense responses to other organisms, to the antimicrobial humoral response, and to the response to virus (**Figure 2A**; **Table S2**). The KEGG enrichment analysis showed that DEGs were mainly associated with viral proteins interacting with cytokines and cytokine receptors, cytokine–cytokine receptor interactions, and the interleukin (IL)-17 signaling pathway (**Figure 2B**) (**Table S3**).

To screen for hub genes, we first constructed a protein–protein interaction (PPI) network with 32 nodes and 1347 interaction edges (**Figure 2C**). In the PPI network, a total of 11 genes (RSAD2, IFIT1, MX1, ISG15, IFI27, OAS3, OAS2, IFI6, IRF7, OAS1, and IFI44L) were identified as hub genes with degrees ≥ 10 (**Table S4**). All of those genes were AVPs, and were significantly upregulated in psoriatic lesions (**Figure 2D**).

### GSEA of scRNA-seq

To investigate the expression of AVPs in psoriatic lesions at single-cell resolution, we collected tissue from 3 psoriasis patients and tissue of a similar region from 3 healthy donors and performed single-cell RNA sequencing (scRNA-seq). After standard data processing and quality control procedures, we obtained transcriptomic profiles for 24,234 cells. Principal cell clusters were classified using an unsupervised graph-based clustering strategy. Cells with similar profiles were annotated using the singleR package.

**Figure 3:**
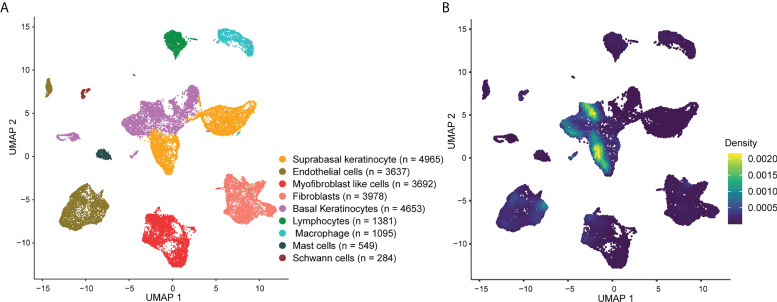
Overview of the single-cell landscape for psoriatic lesions. **(A)** A UMAP view and clustering analysis of combined single-cell transcriptome data from psoriasis and normal skin (*n* = 24234). Clusters are distinguished by different colors, with the general identity of each cell cluster shown on the right. **(B)** Density scatterplot of AVP gene set. UMAP, uniform manifold approximation and projection; AVP, antiviral protein.

The cellular composition is shown in **Figure 3A**. Single-cell rank-based gene set enrichment analysis (GSEA) showed that the enrichment score was higher in keratinocytes (including suprabasal keratinocyte and basal keratinocyte) (**Figure 3B**). The AVP expression levels in cell populations of the psoriatic lesions and normal skin are shown in **Figure S2**. ISG15, MX1, IFI44L, and IFI27 were the characteristic psoriasis genes found in suprabasal keratinocytes (**Table S5**).

### Use of scWGCNA to screen the cytokines correlated with AVP expression

**Figure 4:**
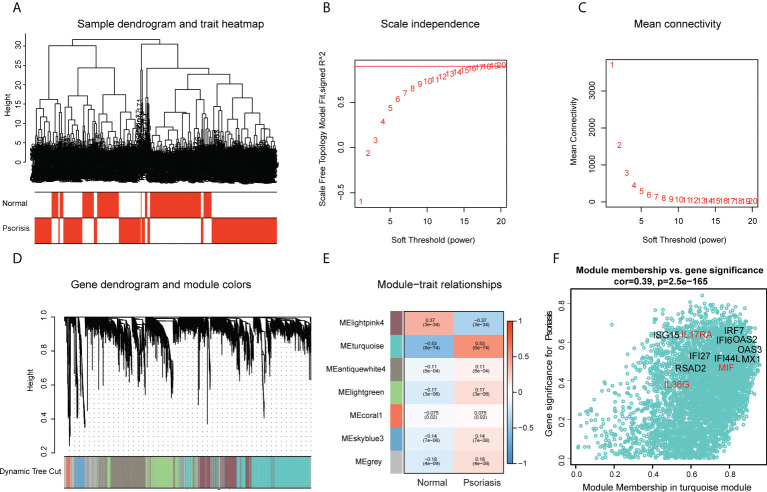
Construction of co-expression modules by the scWGCNA. **(A)** Clustering dendrogram of samples with trait heatmap. **(B)** The relationship between the scale-free fit index and various soft-thresholding powers. **(C)** The relationship between the mean connectivity and various soft-thresholding powers. **(D)** The cluster dendrogram of genes in GSE162183. Branches of the cluster dendrogram of the most connected genes gave rise to 7 gene co-expression modules. **(E)** Heatmap of the correlation between module eigengenes and clinical phenotype (normal and psoriasis). **(F)** Scatter plot describing the relationship between MM and GS in the turquoise module.

The scWGCNA package was used to merge 200 cells into a metacell, which was then used to screen module genes associated with psoriasis in the dataset (GSE162183). A dendrogram of samples with clinical traits were clustered using the average linkage method and Pearson’s correlation method (**Figure 4A**). We determined the soft threshold of 16 by calculating the scale-free model fit and mean connectivity (**Figures 4B, C**). Different module genes in the dynamic tree cut were re-clustered through a topological similarity strategy. Eventually, seven modules developed after merging, and the turquoise module was found to be strongly correlated with psoriasis (**Figure 4D**). We then analyzed the correlation of each module with two clinical traits (normal and psoriasis). We found that AVPs were present in the turquoise module (cor = 0.39, *p* = 2.5e^−165^) (**Figure 4E**) and therefore extracted cytokines from that module (**Figure 4F**; **Table S6**). We obtained three cytokines (MIF, IL17RA, and IL36G) and analyzed the relationship between those cytokines and the AVPs. Only interleukin-36 gamma (IL36G) was found to positively correlate with AVPs (**Table S7**).

### High levels of AVPs and IL36G in suprabasal keratinocytes

**Figure 5:**
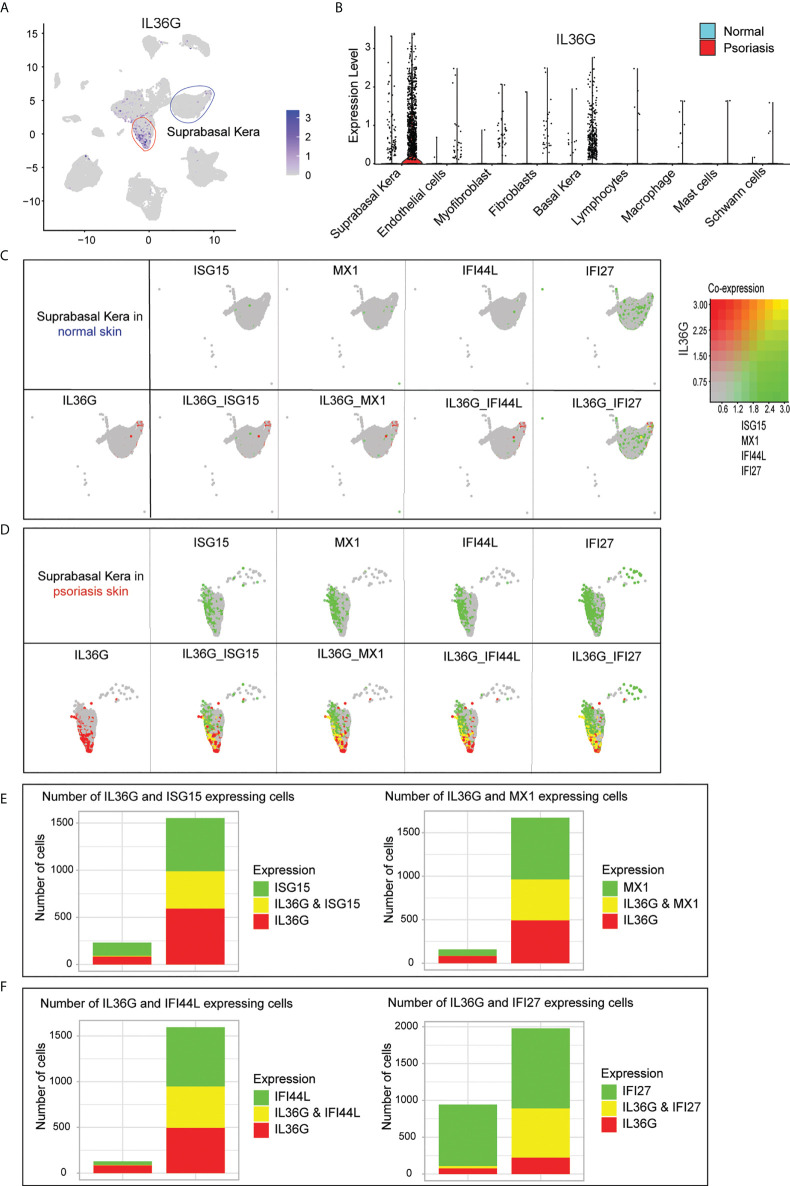
Expression of IL36G and AVPs in suprabasal keratinocytes of psoriatic lesions. **(A)** IL36G expression in total scRNA-seq data is visualized in low-dimensional space. **(B)** Violin plot of each cell cluster. **(C)** Co-expression of IL36G and AVPs in normal skin keratinocytes. **(D)** Co-expression of IL36G and AVPs in suprabasal keratinocytes of psoriatic lesions. **(E, F)** Cells with expression of ISG15, MX1, and IL36G **(E)** and IFI44L, IFI27, and IL36G **(F)** within suprabasal keratinocytes in normal and psoriatic skin are quantified by number of cells.

Of all the IL36G-expressing cells in the scRNA-seq data, IL36G-expressing cells were mainly found in suprabasal keratinocytes (**Figure 5A**). IL36G was upregulated in psoriatic skin compared with normal skin (**Figure 5B**). The IL36G-expressing suprabasal keratinocytes from normal skin did not express any AVPs (**Figure 5C**). The IL36G that was highly expressed in the suprabasal keratinocytes from the psoriatic lesions was also highly co-expressed with AVPs (**Figure 5D**). Suprabasal keratinocytes from psoriatic lesions expressed more AVPs and IL36G than suprabasal keratinocytes from the control skin (**Figures 5E, F**).

### Expression of AVPs and IL36G in patients with psoriasis before and after therapy

**Figure 6:**
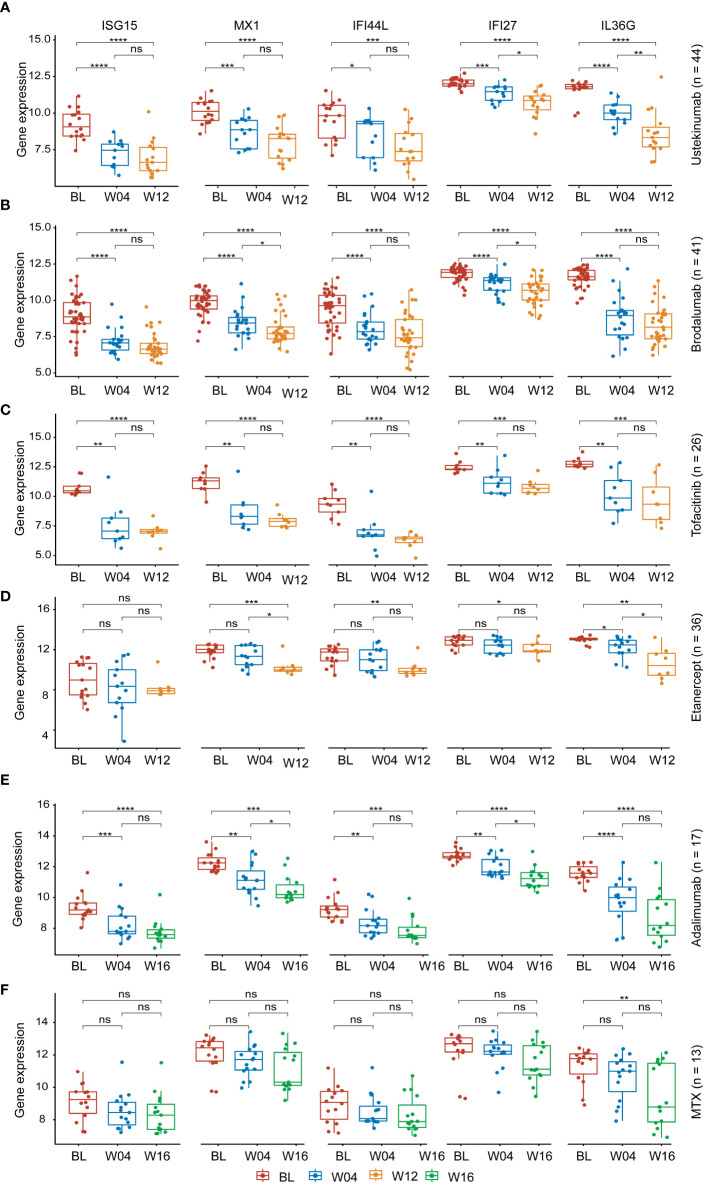
Expression of AVPs and IL36G during treatment. **(A)** Expression of AVPs and IL36G during ustekinumab treatment. **(B)** Expression of AVPs and IL36G during brodalumab treatment. **(C)** Expression of AVPs and IL36G during tofacitinib treatment. **(D)** Expression of AVPs and IL36G during etanercept treatment. **(E)** Expression of AVPs and IL36G during adalimumab treatment. **(F)** Expression of AVPs and IL36G during MTX treatment. MTX, methotrexate. p values were calculated using a Wilcoxon signed-rank test. Asterisks corresponding to p values<0.05 (*< 0.05, **< 0.01, ***< 0.001, ****< 0.0001) and those with p values> 0.05 as not significant "ns" (stat_compare_means function).

The expression levels of the AVPs and IL36G in psoriatic lesions were significantly decreased during treatment, especially at 12 weeks or 16 weeks of treatment. The downward trend was even more pronounced with biologic therapies (adalimumab, ustekinumab and brodalumab, tofacitinib, etanercept) than with MTX (**Figures 6A–F**). We therefore conclude that biological therapy is superior to other drugs in reducing the level of AVPs in psoriasis.

### The correlation between AVPs, IL36G expression levels, and psoriasis area severity index scores during therapy

**Figure 7:**
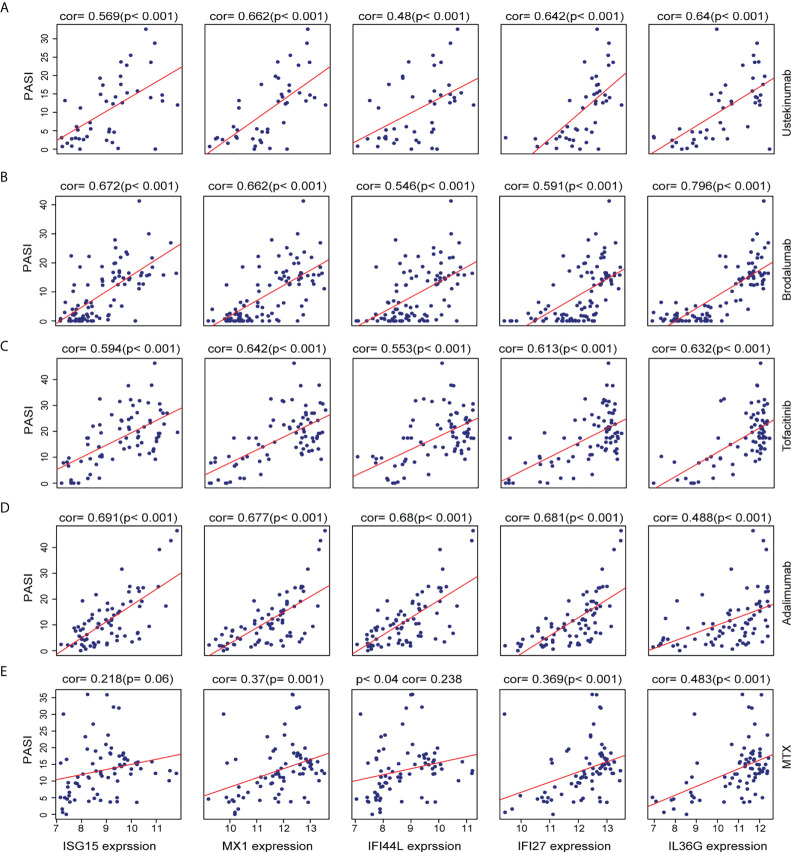
**(A–E)** The relationship between the expression of three biomarkers and PASI scores during psoriasis treatment. **(A)** Ustekinumab treatment. **(B)** Brodalumab treatment. **(C)** Tofacitinib treatment. **(D)** Adalimumab treatment. **(E)** Methotrexate treatment.

In order to explore the association between the AVPs and IL36G expression levels in the context of psoriasis severity, we further investigated the relationship between levels of AVPs and IL36G and Psoriasis Area Severity Index (PASI) scores during psoriasis treatment. Levels of AVPs and IL36G were positively correlated with PASI scores during treatment with adalimumab, ustekinumab, brodalumab, MTX, and tofacitinib. These results therefore suggest that AVPs and IL36G play a role in the severity of psoriasis (**Figures 7A–E**).

## Discussion

It is well accepted that infection with many different microorganisms or viruses, including β-haemolytic streptococci, *Staphylococcus aureus*, *Po`rphyromonas gingivalis*, *Candida albicans*, *Chlamydia psittaci*, HIV, and hepatitis C virus, is a common trigger or exacerbation factor for psoriasis (12). In particular, acute guttate psoriasis is generally thought to be caused by bacterial infection, such as streptococcal infection (13). Beyond the classical role of T helper cell 1 (Th1)/Th2 homeostasis and the IL17/IL23 axis, recently described AMPs have been found to play a role in the immunopathogenesis of psoriasis, further confirming the relationship between infection and psoriasis (4). Numerous high-throughput sequencing studies have suggested that the skin microbiome could play a role in the pathogenesis and therapeutic effect monitoring of psoriasis (14–16) and that AMPs could modify host microenvironments and regulate the colonization of microorganisms (17). It has been suggested that increased expression levels of AMPs could be involved in the pathogenesis of psoriasis (18). AMPs and AVPs are two important functional protein clusters mediating innate immune in psoriatic epidermis. However, the role of AVPs in the keratinocytes of psoriatic lesions remains unelucidated.

In this study, a series of bioinformatics analyses was performed on microarray and single-cell gene expression datasets. We found that the DEGs were mainly associated with a defense response to other organisms, with the type I interferon (IFN) signaling pathway, with the interactions between viral proteins and cytokines, and with the cytokine receptor and cytokine–cytokine receptor interaction pathway. The hub genes were AVPs (RSAD2, IFIT1, MX1, ISG15, IFI27, OAS3, OAS2, IFI6, IRF7, OAS1, and IFI44), which were highly expressed within the keratinocytes in psoriatic lesions. Moreover, IL36G may interplay with ISG15, MX1, IFI44L, and IFI27 in the execution of antiviral function within suprabasal keratinocytes.

Recent findings have demonstrated that the genes of AVPs were overexpressed in psoriatic lesions (8, 10, 19). Zhou et al. found that the keratinocyte-derived OAS2 expression level was positively correlated with PASI scores and decreased after therapy (8). Moreover, in TNF-α–induced HaCaT cells, ISG15 and MX1 were significantly up-regulated (9). Within this series of AVP genes, Mx proteins, such as MX1, are known for inhibiting negative-strand RNA viruses (4, 20).

Interferon-stimulated genes (ISGs) are cellular products that mediate the type I interferon response against a wide range of invading viruses. Only a few ISGs, such as ISG15, IRF7, IFIT1, IFI27, IFI6, and IFI44L, have antimicrobial activity (21). ISG15 (interferon-stimulated gene product 15) participates in many antiviral signaling pathways to directly promote viral clearance (22). IFI27 (interferon α–inducible protein 27) is involved in the proliferation of skin keratinocytes, both in imiquimod-induced psoriasis-like skin and in HaCaT cells (23). Although extensive research confirms that the ISG family are involved in various bacterial and viral infections, the novel functions of AVPs in psoriatic keratinocytes need to be thoroughly investigated.

Although T cells are currently considered to be the main driver of psoriasis, keratinocytes play an important role in regulating inflammation and relapse (24). Epidermal keratinocytes secrete antimicrobial peptides, LL-37, and cytokines like TNF-α, IL6, and IL36 to activate Th1/Th17 cells, which interact with each other to regulate the proliferation and differentiation of keratinocytes (25). IL36G is a pro-inflammatory cytokine whose role in the pathogenesis of psoriasis has been extensively described (26–29). More noteworthy is that our findings showed that AVP genes were abundantly co-expressed with IL36G on psoriatic keratinocytes. Previous research has suggested that IL36G may be an alarmin that signals viral infection (30). One study suggested that IL36G itself is a novel antiviral protein involved in the defense against influenza virus (28). Infectious agents may induce the primary skin barrier layers to produce large amounts of AVPs as a response. Another study found that Th17 cells derived IL29, but not type I IFN-mediated psoriatic keratinocyte is responsible for the expression of the AVPs (31). Whether upregulation of IL36G and AVPs expression in keratinocytes trigger psoriasis is currently unknown.

In addition, the expression levels of AVPs and IL36G were significantly positively correlated with PASI scores, and these levels decreased dramatically during biological treatment. These observations indicate that IL36G and AVPs may be related to the severity of psoriasis and could be used as an indicator for therapeutic efficacy. Research conducted by D’Erme et al. (26) also found that IL36G was closely associated with the disease activity of psoriasis. However, the exact production mechanism of IL36G and AVPs, and the regulatory relationship between them in the context of psoriasis, have still not been sufficiently investigated.

In summary, we demonstrated that the presence of IL36G may mediate the keratinocyte expression of AVPs such as MX1, ISG15, IFI27, and IFI44L in psoriasis vulgaris. The co-expression of AVP genes and IL36G was associated with psoriasis severity and therapeutic efficacy. Our results also suggest that infection factors and AVPs produced by keratinocytes may play an important role in the pathogenesis of psoriasis.

## Data availability statement

The datasets presented in this study can be found in online repositories. The names of the repository/repositories and accession number(s) can be found in the article/**Supplementary Material**.

## Author contributions

Y-WL, H-MW, and Y-JC performed the data analysis and interpreted the results. NS and L-HY prepared the draft. R-JD, Y-QK, and Y-YL designed the research and revised the draft. All authors contributed to the article and approved the submitted version.

## Funding

This work was supported in part by the National Natural Science Foundation of China (81860553), the Talent Introduction Project of Hubei Polytechnic University (21xjz33R, 21xjz34R), the “Ten-thousand Talents Program” of Yunnan Province (YNWR-MY-2018-039), the Project of AIDS Bureau of Yunnan Province, the Yunnan Province Clinical Center for Skin Immune Diseases (ZX2019-03-02), and the Yunnan Province Clinical Research Center for Skin Immune Diseases (2019ZF012).

## Conflict of interest

The authors declare that the research was conducted in the absence of any commercial or financial relationships that could be construed as a potential conflict of interest.

## Publisher’s note

All claims expressed in this article are solely those of the authors and do not necessarily represent those of their affiliated organizations, or those of the publisher, the editors and the reviewers. Any product that may be evaluated in this article, or claim that may be made by its manufacturer, is not guaranteed or endorsed by the publisher.
