# Corrigendum: IL36G is associated with cutaneous antiviral competence in psoriasis

**DOI:** 10.3389/fimmu.2022.1043240

**Published:** 2022-10-17

**Authors:** You-Wang Lu, Yong-Jun Chen, Nian Shi, Lu-Hui Yang, Hong-Mei Wang, Rong-Jing Dong, Yi-Qun Kuang, Yu-Ye Li

**Affiliations:** ^1^ Department of Dermatology and Venereology, First Affiliated Hospital of Kunming Medical University, Kunming, China; ^2^ Hubei Provincial Key Laboratory of Occurrence and Intervention of Kidney Diseases, Medical College, Hubei Polytechnic University, Huangshi, China; ^3^ Department of Dermatology, Huangshi Central Hospital, Affiliated Hospital of Hubei Polytechnic University, Edong Healthcare Group, Huangshi, China; ^4^ NHC Key Laboratory of Drug Addiction Medicine, First Affiliated Hospital of Kunming Medical University, Kunming Medical University, Kunming, China; ^5^ Scientific Research Laboratory Center, First Affiliated Hospital of Kunming Medical University, Kunming, China

**Keywords:** psoriasis, antiviral protein (AVP), IL36G, keratinocyte, treatment

In the published article, there was an error in the author list. You-Wang Lu and Yong-Jun Chen contributed equally to this work and share first authorship. The corrected author list appears below.

You-Wang Lu ^1,2†^, Yong-Jun Chen ^3†^, Nian Shi ^3^, Lu-Hui Yang ^1^, Hong-Mei Wang ^1^, Rong-Jing Dong ^1,2,3*^, Yi-Qun Kuang ^4,5*^, and Yu-Ye Li ^1*^

†These authors have contributed equally to this work and share first authorship

In the published article, there is an error in the title. Instead of “L36G is associated with cutaneous antiviral competence in psoriasis”, it should be “IL36G is associated with cutaneous antiviral competence in psoriasis”.

The authors state that these errors do not change the scientific conclusions of the article in any way. The original article has been updated.

## Publisher’s note

All claims expressed in this article are solely those of the authors and do not necessarily represent those of their affiliated organizations, or those of the publisher, the editors and the reviewers. Any product that may be evaluated in this article, or claim that may be made by its manufacturer, is not guaranteed or endorsed by the publisher.

