# Supplementary figures and images for: IL36G is associated with cutaneous antiviral competence in psoriasis
Source: Front Immunol. 2022 Sep 12;13:971071. doi: 10.3389/fimmu.2022.971071 (PMC9510771; doi:10.3389/fimmu.2022.971071)

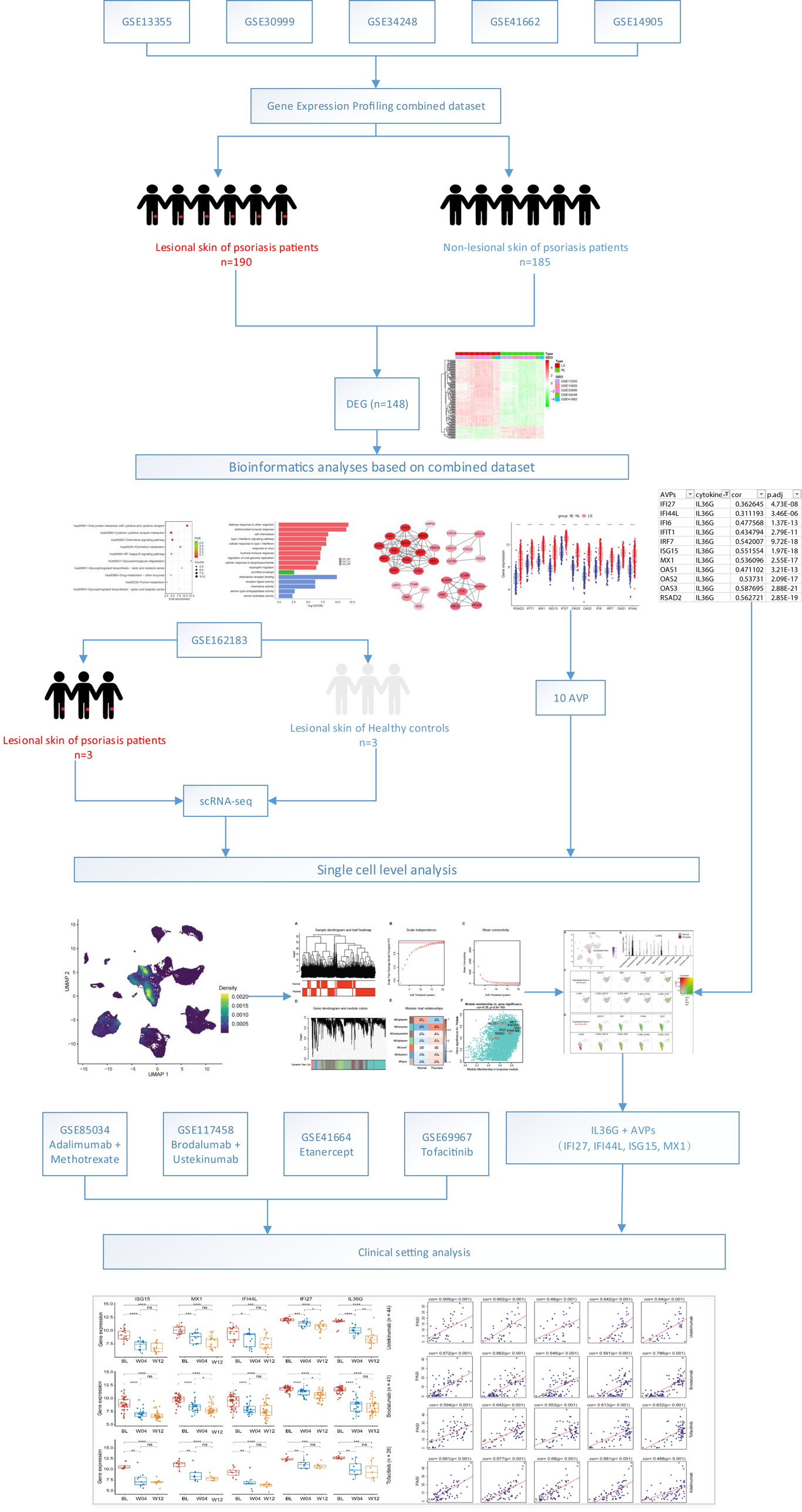

Supplement: Supplementary file 1 [file Image_1.tif]

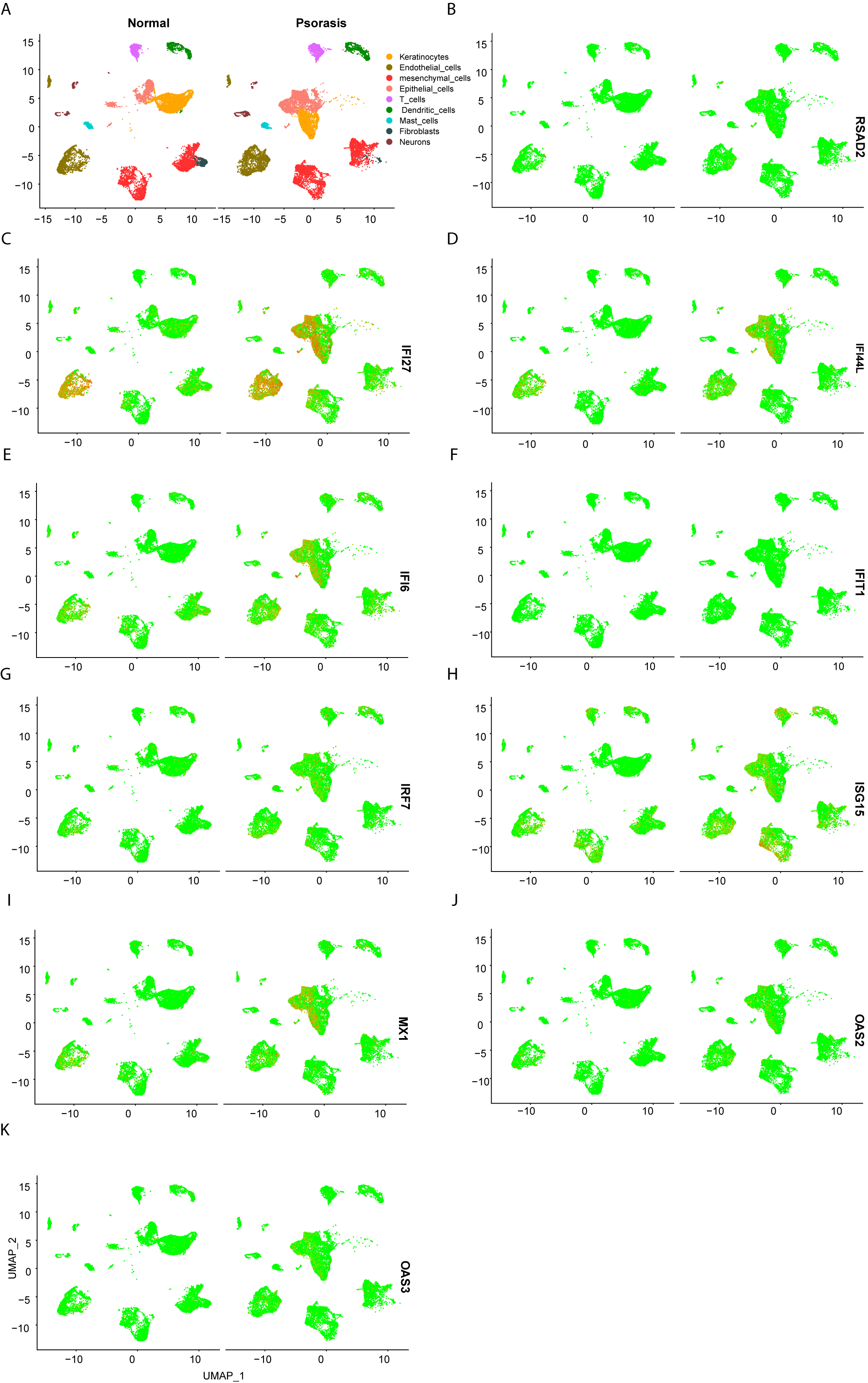

Supplement: Supplementary file 2 [file Image_2.tif]
